# Scheduling radiotherapy for patients with nasopharyngeal carcinoma in the corresponding time window can reduce radiation‐induced oral mucositis: A randomized, prospective study

**DOI:** 10.1002/cam4.6252

**Published:** 2023-08-03

**Authors:** Jun Lv, Shibin Liao, Bo Li, Linjiang Pan, Rensheng Wang

**Affiliations:** ^1^ Department of Radiotherapy The First Affiliated Hospital of Guangxi Medical University Nanning China

**Keywords:** cell cycle, nasopharyngeal carcinoma (NPC), oral mucositis, radiotherapy

## Abstract

**Background:**

To explore a new method to reduce radiation‐induced oral mucositis by scheduling radiotherapy for patients with nasopharyngeal carcinoma (NPC) in the corresponding time window of the cycle of oral mucosal cells.

**Methods:**

Eighty‐two NPC patients were randomly divided into a day group (*n* = 41) and a night group (*n* = 41). The radiotherapy was scheduled at noon (11:30–15:30) for the day group, while at night (19:00–23:00) for the night group. Oral mucositis and oral pain were recorded in both groups after each radiotherapy fraction. The short‐term efficacy of primary tumor regression, weight loss, and bone marrow suppression was recorded.

**Results:**

The incidence of Grade 2 oral mucositis was 87.8% (36/41) and 63.4% (26/41) in the night group and day group, respectively (*p* = 0.010). The incidence of Grade 3 oral mucositis was 65.9% (27/41) and 22.0% (9/41) in the night group and day group, respectively (*p* < 0.001). The mean number of radiotherapy for patients to develop Grade 2 oral mucositis was 15.67 ± 5.05 and 20.92 ± 6.21 in the night group and day group, respectively. The incidence of Grade 2 oral pain was 48.8% (20/41) and 22.0% (9/41) in the night group and day group, respectively (*p* = 0.011). There were no significant differences in tumor regression, weight loss, and bone marrow suppression between the two groups.

**Conclusion:**

By scheduling radiotherapy based on the corresponding time window of the cycle of oral mucosal cells, the severity of oral mucositis in NPC patients was reduced.

## 1 INTRODUCTION

Radiotherapy serves as the main therapy for nasopharyngeal carcinoma (NPC). However, radiation‐related toxicities cannot be avoided, for example, oral mucositis during radiotherapy is a common complication in NPC patients.^1^ Severe oral mucositis may reduce treatment compliance, and even lead to the discontinuation of radiotherapy, thus affecting the therapeutic efficacy. Studies have shown that the severity of oral mucositis might be influenced by the cell cycle.^2^ In the proliferating tissues of mammals, particularly in the oral mucosa of humans, the diurnal variation in the cell cycle is an important determinant of radiosensitivity.^3^

The normal operation during a cell cycle is mainly regulated by cyclins, protein‐dependent kinases, and protein‐dependent kinase inhibitors.^4^ In many tissues, cyclins involved in cell cycle progression, cell proliferation, and apoptosis show circadian rhythms.^5^ Bjarnason et al.^6^, ^7^ found that the protein parameters of human oral mucosal cells peaked in the Gap1 phase (G1 phase) at about 11:00, the DNA synthesis phase (S phase) at 15:00, in Gap2 phase (G2 phase) at 16: and in mitotic phase (M phase) at 21:00. It is the most radiosensitive in the G2 phase and M phase, the lowest in the S phase, and medium in the G1 phase.^8^

Therefore, we designed a prospective randomized controlled study to observe the severity of oral mucositis and oral pain caused by radiotherapy in a corresponding time window of different cycles of oral mucosal cells and explore a new method to reduce radiation‐induced oral mucositis in NPC patients.

## 2 METHODS AND MATERIALS

### 2.1 Study design

The study, as a prospective randomized clinical trial, aims to compare the severity of oral mucositis and the degree of oral pain in NPC patients treated with radiotherapy at different time points during the cycle of oral mucosal cells. The clinical trial was approved by the Ethics Committee of the First Affiliated Hospital of Guangxi Medical University and successfully registered with the China Clinical Trial Registry (ChiCTR2100047661). All the patients signed an informed consent form before treatment was started.

### 2.2 Patients

A total of 82 treatment‐naive NPC patients, who were diagnosed by pathological biopsy in our hospital from August 2019 to December 2020, were included in this study. The inclusion criteria include treatment‐naive NPC patients with a Karnofsky Performance Status (KPS) score ≥ 70 points; patients who received intensity‐modulated radiation therapy (IMRT); patients with normal oral mucosa; patients who did not receive other anti‐tumor treatment before radiotherapy or chemotherapy, patients without serious diseases of the heart, lung, liver, and kidney; and patients who voluntarily signed the informed consent. The exclusion criteria include patients with abnormal sleep habits, oral mucosal injury, or oral mucosal diseases which may affect observation before radiotherapy in case of active infection; patients with a bleeding tendency or immune diseases; patients who lack self‐thinking and expression ability; patients who are unable to complete oral scoring criteria; and patients with severe bone marrow dysfunction.

The eighth edition criteria issued by the Union for International Cancer Control (UICC) were referred for clinical staging in the study.

All the patients underwent dental evaluation before radiotherapy, and nasopharyngoscopy was completed for all of them. Their imaging examinations included enhanced MRI of the nasopharynx and cervical soft tissues, chest CT, whole‐body bone scan, and abdominal ultrasound or abdominal CT. If required, whole‐body PET/CT would be performed. Hematological evaluation (platelet count ≥100,000/μL, hemoglobin ≥10 gm/dL, and absolute neutrophil count ≥1500/μL), liver and kidney function evaluation (serum bilirubin, alanine aminotransferase, aspartate aminotransferase equal to or 1.5 times higher than the upper limit of normal, endogenous creatinine clearance ≥60 mL/min) would be performed.

### 2.3 Radiotherapy

#### 2.3.1 Delineating target volume

Gross tumor volume of nasopharyngeal carcinoma (GTVnx) refers to the area of primary tumors in the nasopharynx observed in clinical practice and various imaging studies. Gross tumor volume of cervical node (GTVnd) refers to the area of enlarged lymph nodes observed in clinical practice and/or imaging studies. The clinical target volume (CTV) range is mainly divided into high‐ and low‐risk areas according to the local progression of NPC. Clinical target volume 1 (CTV1) is defined as the adjacent area around a primary tumor that is very likely to be invaded by the tumor or the area to which the tumor is very likely to metastasize. Clinical target volume 2 (CTV2) is defined as a lymph node region with possible metastasis deduced from the biological behavior of a tumor, including the cervical lymphatic drainage area without metastatic lymph nodes.^9^, ^10^

Organs at risk (OAR) should be delineated by including the brainstem, cervical spinal cord, temporal lobe, optic nerve, optic chiasm, pituitary gland, lens, temporomandibular joint, mandible, inner ear, and parotid gland.^11^

Oral delineation ranged from the hard palate to the floor of the mouth, from the buccal mucosa around the teeth to the lingual surface and uvula.^12^

#### 2.3.2 Preparing dose prescription, and submitting, designing, and evaluating plan

All patients received IMRT. The prescribed dose was 68–72 Gy, 68–70 Gy, 60–62 Gy, and 54–56 Gy, in 30–32 fractions, for the planning target volumes derived from GTVnx, GTVnd, CTV1, and CTV2, respectively. QUANTEC (2012 Standard) was referred for limiting dose to OAR.^12^ What's more, there was Dmean≤40Gy for the oral cavity.

#### 2.3.3 Radiotherapy schedule

Eighty‐two NPC patients were randomly divided into two groups, a day group, and a night group, according to the corresponding period of different cycles of human oral mucosal cells during a day. Radiotherapy was scheduled for the patients in the two groups between 11:30 ~ 15:30 (S phase) at noon and 19:00 ~ 23:00 (M phase) at night, respectively.

### 2.4 Chemotherapy

A chemotherapy regimen might be concurrent chemotherapy or induction chemotherapy plus concurrent chemotherapy. In this study, the observation of oral mucositis was terminated at the end of radiotherapy. All patients used a 3‐week regimen of platinum‐based drugs. Chemotherapy was suspended if severe hematological toxicity or hepatorenal function impairment occurred during treatment. Symptomatic and supportive treatment was performed in time for acute toxicity. The treatment plan was delayed or stopped according to the patient's condition, if necessary.

### 2.5 Evaluation criteria

#### 2.5.1 Scoring mucosal injury

According to the grading criteria for acute mucosal reactions in the Evaluation Criteria for Acute Radiation Injury, issued by the Radiation Therapy Oncology Group (RTOG),^13^ the grade of oral mucositis of the patients was recorded.

#### 2.5.2 Grading pain response

According to the Verbal rating scale (VRS),^14^ oral pain grading was recorded.

### 2.6 Management of oral mucositis

After agreeing to join the study, the patients were asked to keep oral hygiene, brush their teeth in the morning and evening, and rinse their mouths after each meal. Once the radiotherapy was started, the patients should gargle with Compound Borax Solution/baking soda daily. Non‐steroidal anti‐inflammatory drugs were given for pain relief as oral pain worsened. Radiotherapy should be suspended if there were Grade 4 oral mucosal reactions.

### 2.7 Statistical analysis

In this study, SPSS 23.0 was used for the statistical analysis of the data. All normal measurement data were expressed as mean ± standard deviation, and the independent normal and equal variance data of the two groups were compared with the t‐test of two independent samples. All the computing data were expressed as case numbers and percentages. The chi‐square test was used for comparison between the two groups, and the continuous correction chi‐square test was adopted when there was 1 < theoretical frequency (T) < 5. The test level α = 0.05 was taken in the statistical analysis.

## 3 RESULTS

### 3.1 Baseline data of patients

A total of 82 NPC patients diagnosed in our hospital were included in this study. Of them, 62 (75.6%) were male and 20 (24.4%) females, with an average age of 48.29 ± 12.62 years. There were 12 patients in stages I and II, and 70 in stages III and IV. Their chemotherapy regimen was induction chemotherapy plus concurrent chemotherapy or concurrent chemotherapy, and the pathological type was undifferentiated non‐keratinizing carcinoma for most of the patients. The differences in the general data had no statistical significance (*p* > 0.05), but were comparable between the two groups, as shown in Table 1.

**TABLE 1:** Basic data statistics of research objects.

Group	Night group (n = 41)	Day group (n = 41)	χ^2^/*t*	*p‐Value*
Sex				
Male	34	28	2.381	0.123
Female	7	13		
Age				
Mean age	49.32 ± 12.71	47.27 ± 12.60	0.733	0.466
< 50 years	19	23	0.781	0.377
≥ 50 years	22	18		
Clinical stage				
Stage I–II	6	6	0.514	0.773
Stage III	16	19		
Stage IV	19	16		
Exposure dose (Gy)				
Primary tumor	74.16 ± 0.91	74.17 ± 1.74	0.056	0.956
Oral cavity	38.60 ± 2.98	38.18 ± 3.53	0.589	0.558
Chemotherapy regimen				
Concurrent chemotherapy	9	8	0.074	0.785
Induction chemotherapy plus concurrent chemotherapy	32	33		
Histopathological features				
Undifferentiated non‐keratinizing carcinoma	39	35	1.247	0.264
Differentiated non‐keratinizing carcinoma	2	6		
Smoking history				
Yes	24	16	3.124	0.077
No	17	25		

### 3.2 Comparison of the severity of oral mucositis

In the night group, 41 patients reported Grade 1 oral mucositis, 36 ones Grade 2 oral mucositis, and 27 ones Grade 3 oral mucositis during the radiotherapy; the incidence of Grade 2 and 3 oral mucositides was 87.8% (36/41) and 65.9% (27/41), respectively. In the day group, 41 patients reported Grade 1 oral mucositis, 26 ones Grade 2 oral mucositis, and 9 ones Grade 3 oral mucositis during the radiotherapy; the incidence of Grade 2 and 3 oral mucositides was 63.4% (26/41) and 22.0% (9/41), respectively. The incidence of Grade 2 and 3 oral mucositides was lower in the day group than that in the night group, and the differences were statistically significant (*χ* ^2^ = 6.613, *p* = 0.010),(*χ* ^2^ = 16.043, *p* < 0.001). No patient reported Grade 4 oral mucositis in either group. Please refer to Table 2 for more information.

**TABLE 2:** Incidence of acute oral mucositis during radiotherapy in the two groups (*n*, %).

Group	Number of patients	Grade of oral mucositis			
		1	2	3	4
Night group	41	41 (100%)	36 (87.8%)	27 (65.9%)	0 (0%)
Day group	41	41 (100%)	26 (63.4%)	9 (22.0%)	0 (0%)

### 3.3 Comparison of the onset time of oral mucositis

**FIGURE 1:**
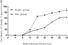
Cumulative incidence of Grade 2 mucositis in the night and day groups during radiotherapy.

**FIGURE 2:**
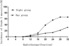
Cumulative incidence of Grade 3 mucositis in the night and day groups during radiotherapy.

As the exposure dose was increased in the radiotherapy, the cumulative incidence of Grade 2 and 3 oral mucositis gradually increased in the patients of the two groups (as shown in Figure 1 and Figure 2).

The mean value of radiotherapy for the patients to develop Grade 2 oral mucositis was 15.67 ± 5.05 and 20.92 ± 6.21 in the night group and the day group, respectively. Compared with the night group, the patients needed more exposure to develop Grade 2 oral mucositis in the day group, which also meant that the time to develop Grade 2 oral mucositis was significantly delayed for the day group. Their difference was statistically significant (*t* = 3.672, *p* = 0.001). However, the mean value to develop Grade 3 oral mucositis was 21.00 ± 4.25 for the night group and 21.44 ± 6.69 for the day group. The difference was not statistically significant between the two groups (*t* = 0.234, *p* = 0.816).

### 3.4 Comparison of oral pain severity

Forty patients reported Grade 1 oral pain, 20 ones Grade 2 oral pain, and 2 ones Grade 3 oral pain in the night group so that their incidence of Grade 2 and 3 oral pain was 48.8% (20/41) and 4.9% (2/41), respectively; while 40 patients reported Grade 1 oral pain, 9 ones Grade 2 oral pain, and 1 one Grade 3 oral pain in the day group so that their incidence of Grade 2 and 3 oral pain was 22.0% (9/41) and 2.4% (1/41), respectively. The incidence of Grade 2 oral pain was lower in the day group compared with the night group, and the difference was statistically significant (*χ* ^2^ = 6.455, *p* = 0.011). However, there was no statistical significance in the incidence difference of Grade 3 oral pain between the two groups (χ^2^ = 0.000, *p* = 1.000). Please see Table 3 for more details.

**TABLE 3:** Analysis of the onset of oral pain in the two groups during radiotherapy (*n*, %).

Group	Number of patients	Pain level		
		1	2	3
Night group	41	40 (97.6%)	20 (48.8%)	2 (4.9%)
Day group	41	40 (97.6%)	9 (22.0%)	1 (2.4%)

### 3.5 Tumor regression

Within 1 month after the radiotherapy, 3 patients achieved complete response (CR), 36 achieved partial response (PR) and 2 ones achieved stable disease (SD) in the night group, with a CR rate and PR rate of 7.3% (3/41) and 87.8% (36/41), respectively, while five patients achieved CR, 35 ones PR and 1 one SD in the day group, with CR rate and a PR rate of 12.2% (5/41) and 85.4% (35/41), respectively. The overall response rate (ORR = CR + PR) of the patients was 95.1% (39/41) and 97.6% (40/41) in the night group and day group, respectively.

The differences in CR rate, PR rate and ORR were not statistically significant between the night group and day group (*χ* ^2^ = 0.139, *p* = 0.710), (*χ* ^2^ = 0.105, *p* = 0.746), (*χ* ^2^ = 0.000, *p* = 1.000). No patient achieved progressive disease in either of the group. Please see Table 4 for more details.

**TABLE 4:** Evaluation of the curative effect in the two groups within one month after radiotherapy (*n*, %).

Group	Number of patients	CR	PR	SD	PD	OR(CR + PR)
Night group	41	3 (7.3%)	36 (87.8%)	2 (4.9%)	0 (0%)	39 (95.1%)
Day group	41	5 (12.2%)	35 (85.4%)	1 (2.4%)	0 (0%)	40 (97.6%)

### 3.6 Comparison of bone marrow suppression and weight loss

During the radiotherapy, 17 patients experienced Grade 1 bone marrow suppression, 16 ones Grade 2 bone marrow suppression, and 7 ones Grade 3 bone marrow suppression in the night group so that their incidence of Grade 2 and Grade 3 bone marrow suppression was 39.0% (16/41) and 17.1% (7/41), respectively; while 15 ones Grade 1 bone marrow suppression, 13 ones Grade 2 bone marrow suppression, and 11 ones Grade 3 bone marrow suppression in the day group, so that their incidence of Grade 2 and Grade 3 bone marrow suppression was 31.7% (13/41) and 26.8% (11/41), respectively. There was no statistical significance in the incidence difference of Grade 2 and 3 bone marrow suppression between the two groups (χ^2^ = 0.480, *p* = 0.488), (χ^2^ = 1.139, *p* = 0.286). There was one patient who experienced Grade 4 bone marrow suppression during radiotherapy in both groups, respectively. Please see Table 5 for more details.

**TABLE 5:** Onset of bone marrow suppression in the two groups during radiotherapy (*n*,%).

Group	Number of patients	Bone marrow suppression				
		0	1	2	3	4
Night group	41	0 (0%)	17 (41.5%)	16 (39.0%)	7 (17.1%)	1 (2.4%)
Day group	41	1 (2.4%)	15 (36.6%)	13 (31.7%)	11 (26.8%)	1 (2.4%)

The mean weight loss after radiotherapy was 6.38 ± 2.85 kg and 5.53 ± 2.40 kg in the night group and day group, respectively. There was no statistical significance in the difference in such mean weight loss between the two groups (*t* = 1.455, *p* = 0.150).

## 4 DISCUSSION

In clinical practice, the vast majority of NPC patients would experience varying degrees of oral mucositis during radiotherapy, and the incidence of severe radiation‐induced oral mucositis ranges from 20% to 40%.^15^, ^16^, ^17^ Severe oral mucositis may require dose reduction or interruption of radiotherapy for NPC patients, thus affecting their therapeutic effect.^18^ Therefore, it is very important to reduce the severity of oral mucositis for NPC patients during radiotherapy.

The pathogenesis of oral mucositis resulting from concurrent chemo‐radiotherapy is mainly divided into five stages, namely, initiation, upregulation/activation, signal amplification, ulceration, and healing stages.^19^ Due to its complex biological nature, oral mucositis is mainly treated with preventive and symptomatic supportive methods. There are several treatment options available, but strong evidence is still lacking to support the prevention and treatment of oral mucositis.^20^, ^21^, ^22^

Due to the rotation of the earth, organisms constantly cycle during daytime and nighttime, and most of them have their circadian rhythms, which regulate the body's physiological and metabolic processes to adapt to different times in the day.^23^ Previously accumulated research evidence suggests that there is a close interaction between circadian rhythm and cell cycle, another basic rhythmic process, which is bi‐directionally linked to circadian rhythm. Clock genes correspond to different cell cycles at different time windows in a day by participating in the regulation of important cell cycle checkpoints.^24^, ^25^

In this study, based on the cycle of oral mucosal cells, radiotherapy was scheduled in the corresponding time window of the cell cycle according to the different cycle distribution patterns of oral mucosal cells in a day, to explore a new method to reduce radiation‐induced oral mucositis in NPC patients. We found that Grade 2 and 3 oral mucositis caused by radiotherapy at noon (11:30 ~ 15:30) was milder than that caused by radiotherapy at night (19:00 ~ 23:00), and the occurrence time of Grade 2 oral mucositis after radiotherapy conducted at noon was also delayed compared with radiotherapy conducted at night. We also found that the incidence of Grade 2 oral pain caused by radiotherapy conducted at noon was milder than that caused by radiotherapy conducted at night. There was no significant difference in the short‐term efficacy of tumor regression between the day group and night group, nor in the degree of bone marrow suppression or weight loss during treatment.

Bjarnason et al.^26^ randomly divided patients with head and neck cancer into a morning group (8:00 ~ 10:00) and an afternoon group (16:00 ~ 18:00) for radiotherapy. It was found that there was no statistical significance in the incidence difference of Grade 3 oral mucositis among the patients in the afternoon group (*p* = 0.17). However, in a stratified analysis of patients, it was found that among patients whose exposure dose was 66 ~ 70Gy or higher, the incidence of Grade 3 oral mucositis was 44.6% and 67.3% in the morning group and afternoon group, respectively; it was significantly lower in the morning group than that in the afternoon group, and the difference was statistically significant (*p* = 0.022). Meanwhile, the mean occurrence time of Grade 3 oral mucositis was 7.9 weeks and 5.6 weeks in the morning group and afternoon group, respectively; it was later in the morning group than in the afternoon group, and the difference was statistically significant (*p* = 0.033). Goyal et al.^27^ randomly divided patients with head and neck cancer into a morning group (8:00 ~ 11:00) and an afternoon group (15:00 ~ 18:00) and found that the incidence of Grade 3 and 4 oral mucositis was higher in the afternoon group compared with that in the morning group, but the difference was not statistically significant (*p* = 0.08). In terms of radiotherapy time, the most sensitive G2 phase and the relatively insensitive G1 phase were observed in the two studies, but the least sensitive S phase was not. Overall, the result was not significantly different, but there was a significant difference in patient subgroups receiving the dose of 66 ~ 70 Gy or higher. Our findings were similar to those from the NPC patients receiving a mean exposure dose of 74 Gy. In our study, we innovatively chose the most sensitive M phase and the least sensitive S phase for comparison in terms of the selection of radiotherapy time. Therefore, the differences between the two groups were more significant.

Fang Yi et al.^28^ scheduled the radiotherapy time for patients with head and neck cancer at 7‐time intervals (8:30 ~ 16:30) during their standard treatment process. It was found that the radiotherapy time of patients was significantly correlated with the severity of oral mucositis (*p* = 0.02). The results were similar to our findings.

In addition, the cases in previous studies include a variety of diseases, and the anatomical location of the primary tumor is different, which may affect the reliability of the research conclusions. With sufficient disease sources, Guangxi, China is of a high incidence of NPC. In our study, NPC was studied by separately selecting the relatively fixed locations of the primary tumor and relatively the same exposure dose to the oral cavity. At the same time, it was analyzed when there was no significant difference in the exposure dose to the oral cavity between the two groups, so the correlation between radiation‐induced oral mucositis and radiotherapy time window in NPC patients was demonstrated in a comparably better manner.

Oral pain score is generally related to the severity of oral mucositis.^29^ In this study, the incidence of Grade 2 oral pain was lower in the day group than in the night group, indicating that there was a strong correlation between oral pain score and the severity of oral mucositis and that radiotherapy conducted at noon might reduce the incidence of Grade 2 pain by reducing the severity of oral mucositis.

Previous studies have shown that weight loss due to radiotherapy has adverse effects on the prognosis of NPC patients.^30^, ^31^ Our study found that there was no significant difference in the degree of weight loss during radiotherapy between the two groups。Although there were significant differences in the severity of oral mucositis and oral pain between the two groups, the patient's swallowing function, and gastrointestinal digestion, and absorption function were less damaged by radiation in the two groups. What's more, thanks to the nutritional education during treatment, the patients were basically able to follow the doctor's advice to supplement nutrition.

Since bone marrow suppression was mainly caused by chemotherapy and there was no significant difference in the number of patients undergoing concurrent chemotherapy between the two groups, there was no significant difference in the degree of bone marrow suppression between the two groups.

At the same time, we also compared the tumor regression between the two groups, and there was no significant statistical difference in this regard in the two groups, either. This indicates that radiotherapy at the different time points of the cycle of oral mucosal epithelial cells does not affect the short‐term efficacy of cancer treatment.

This study has several limitations. Due to time constraints, we included a small number of cases. Also, due to machine failure, working system, and staffing, we could not ensure that all radiotherapy operations for the patients were scheduled within the predetermined period, but have ensured that over 90% of radiotherapy operations for all the patients were conducted within the predetermined period. We will also count the differences in the relapse rate and overall survival rate of patients between the two groups in the future. In addition, the duration and recovery time of oral mucositis of patients after radiotherapy were not further tracked, therefore, they need to be further explored in future studies.

In summary, our results suggest that by scheduling radiotherapy based on the corresponding time window of the cycle of oral mucosal cells, the severity of oral mucositis and oral pain was reduced in NPC patients during radiotherapy. It neither affected the efficacy nor increase treatment toxicity. Therefore, this radiotherapy mode might be an effective way to reduce radiation‐induced oral mucositis but not increase the cost in NPC patients.

## AUTHOR CONTRIBUTIONS

**Jun Lv:** Conceptualization (lead); project administration (equal); writing – review and editing (lead). **Shibin Liao:** Conceptualization (supporting); data curation (lead); methodology (lead); writing – original draft (equal). **Bo Li:** Data curation (supporting); formal analysis (supporting); resources (supporting); software (supporting). **Linjiang Pan:** Funding acquisition (supporting); project administration (supporting). **Rensheng Wang:** Conceptualization (supporting); project administration (lead); writing – review and editing (supporting).

## FUNDING INFORMATION

Not applicable.

## CONFLICT OF INTEREST STATEMENT

All authors made no disclosures.

## Data Availability

All data, models, and code generated or used during the study appear in the submitted article.
